# Single-Use Bag Valve Masks: Evaluation of Device Design and Residual Bioburden Analytical Methods

**DOI:** 10.4236/jbise.2018.119019

**Published:** 2018-08-29

**Authors:** Sarah Zemitis, Melinda Harman, Zachary Hargett, Donna Weinbrenner

**Affiliations:** 1 Department of Bioengineering, Clemson University, Clemson, USA;; 2 Department of Biological Sciences, Clemson University, Clemson, USA

**Keywords:** Reprocessing, Low-Resource, Bag Valve Mask, Single-Use Device, Low-and Middle-Income Countries, Newborn Resuscitation

## Abstract

**Background:**

A recent survey of in-hospital reprocessing in Tanzanian hospitals identified bag-valve masks (BVM) as a commonly reused single-use device. In low- and middle-income countries (LMIC), in-hospital reprocessing supports neonatal resuscitation strategies by helping to maintain adequate supplies of BVM. However, there is a need for device-specific protocols defining reprocessing procedures and inspection criteria to overcome variations in reprocessing practices between hospitals. The purposes of this study were: 1) to complete a comprehensive design review and identify challenges to reprocessing BVMs; and 2) to investigate three different residual bioburden analysis methods for assessing the efficacy of decontaminating a disposable BVM.

**Methods:**

New, unused bag-valve-masks were contaminated with *Staphylococcus epidermidis* and Artificial Mucus Soil to simulate the worst case soiling conditions. Devices underwent one of five disinfection protocols, including one currently used in a LMIC hospital. Three analytical (two quantitative and one qualitative) methods were selected to evaluate residual bioburden on the device following decontamination.

**Results:**

Of all protocols tested, only the positive control and the Soap and Bleach protocols met disinfection targets. Most cleaning outcomes were consistent from trial to trial for each protocol. However, cleaning outcomes varied greatly for the Alcohol Wipe protocol. For the residual bioburden analyses, the two quantitative methods produced similar results, but the qualitative measurement exhibited increased variability.

**Conclusion:**

While this study revealed positive disinfection outcomes for the Tanzanian hospital decontamination protocol, more studies are required to support these findings. Design features of the BVM mask presented challenges to cleaning and drying during different decontamination protocols, as seen in the variability in the Alcohol Wipe protocol performance. These findings support the case for a device-specific protocol for the BVM. Given proper hospital personnel training and available resources, in-hospital reprocessing could support neonatal resuscitation strategies and other demands for manual resuscitation by helping to maintain adequate supplies of BVM.

## 1. INTRODUCTION

Regulatory agencies define a single-use device (SUD) as a medical device that is designated by the manufacturer for use during a single medical procedure on a single patient and is intended to be discarded after the procedure [1–4]. However, used SUDs are not discarded in all circumstances; rather, they are sometimes reprocessed for reuse using specific methods for cleaning and disinfection. Recent trends indicate regulated reprocessing is often performed by third-party reprocessors who are independent from healthcare facilities [4–8], but in-hospital reprocessing has been reported for many different types of SUDs and remains prevalent in many low- and middle-income countries (LMIC) [9–13].

The current investigation was motivated by a recent survey of in-hospital reprocessing in Tanzanian hospitals that identified bag-valve masks (BVM) as a commonly reused SUD [10]. BVM are medical devices commonly used in intensive care units and other key hospital departments to treat patients requiring ventilation during manual resuscitation [14, 15]. BVM are considered an essential piece of equipment for newborn resuscitation [16–18], as failure to establish breathing accounts for 19% of neonatal deaths and the 3% - 6% of babies born requiring basic resuscitation using a BVM [19–21]. In LMIC with a high burden of neonatal mortality, inadequate supplies and poorly functioning BVM can contribute to inconsistent resuscitation practices [16]. Therefore, well-executed in-hospital reprocessing could support neonatal resuscitation strategies by helping to maintain adequate supplies of BVM.

Recognized challenges with in-hospital reprocessing include variations in reprocessing practices between hospitals and a need for device-specific protocols defining reprocessing procedures and inspection criteria [8, 10, 12, 13, 22, 23]. In the Tanzanian survey [10], hospital personnel reported that BVM were reprocessed using a generalized decontamination protocol consisting of extended exposure to a dilute bleach solution followed by a water rinse and air-drying. However, varied reprocessing methods applied to some SUDs were noted, including use of alcohol wipes and simple water rinsing when devices were perceived as low-risk of contamination [10]. At present, there are limited data available for reprocessing disposable BVM. Manufacturers of reusable BVM propose some methods for decontamination in their instructions for use, but validation data are not provided [24, 25]. Those methods recommend the use of detergents and manual scrubbing for cleaning, the use of glutaraldehyde or sodium hypochlorite solutions for chemical disinfection, and the use of ethylene oxide or steam sterilization for sterilization.

The purposes of the current study were: 1) to complete a comprehensive design review and identify challenges to reprocessing BVMs; and 2) to investigate three different residual bioburden analysis methods for assessing the efficacy of decontaminating a disposable BVM.

## 2. METHODS & MATERIALS

### 2.1. Design Review

BVM designs have basic common features, including a soft polymer mask to conform to the patient’s face, a deformable ventilation bag, a non-rebreathing valve connecting the mask to one end of the bag, and an air intake valve at the opposite end of the bag. Worldwide, self-inflating BVM are the most common manual ventilation device used in neonatal and adult intensive care units [15, 18, 26]. As described by Davies, *et al.* [15], self-inflating BVM are portable and versatile due to their ability to fill with ambient room air or with gas supplied from an external oxygen tank. When the ventilation bag on a self-inflating BVM is compressed, the non-rebreathing valve directs gas from the bag to the patient. As pressure on the bag is released, the non-rebreathing valve closes and gas exhaled by the patient is directed out of the mask through a separate channel in the non-rebreathing valve while the bag automatically re-inflates through the air intake valve.

**Figure 1.**
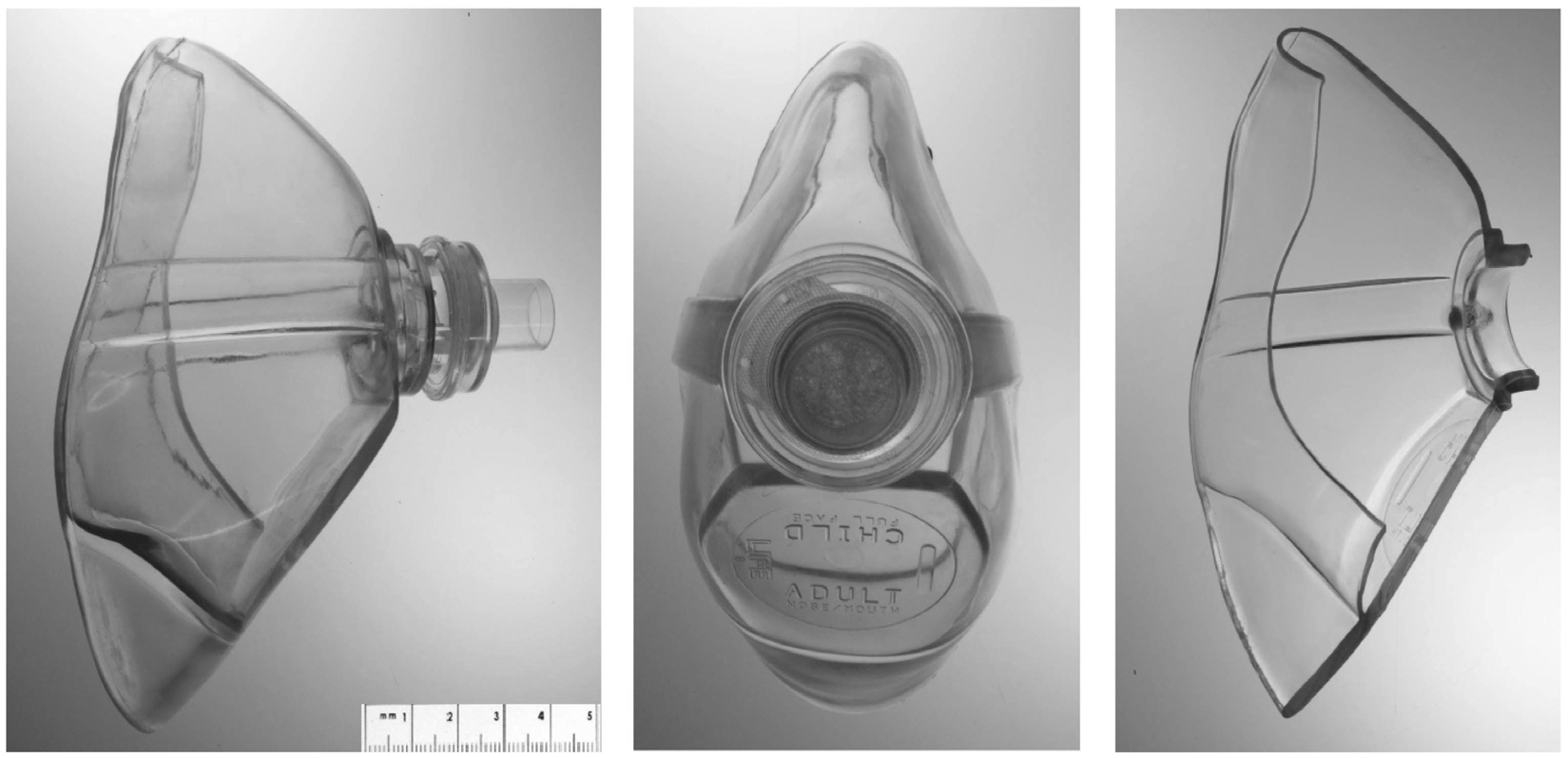
The Test BVM consisted of a pliable facemask and a rigid non-rebreathing valve. All residual bioburden analysis methods were completed on masks that were cut in half after removal of the valve.

BVM can either be reusable or disposable. For the purposes of the current study, disposable BVM, hereafter referred to as Test BVM (Figure 1), were purchased from a commercial source (Model Life-100, Life Corporation, Milwaukee, WI). According to specifications provided by the manufacturer, the Test BVM consisted of a clear face mask fabricated from a thermoplastic polymer (polyvinyl chloride) with a removable rigid plastic one-way valve housing a hydrophobic filter (Filtrete, 3M Corporation, St. Paul, MN). This mask features a 15-mm diameter air intake opening and a hydrophobic filter above the valve to protect BVM components from body fluids.

Several design features of the Test BVM were considered reprocessing challenges, including small crevices near the valve attachment, contours on the outside surface, and tight folds inside the mask (Figure 1). These features are opportunistic areas for bacteria and physical debris buildup. The mask is made of a pliable material, which can add to the challenge of reprocessing [27]. Considering regulatory demands for worst-case contamination conditions [1], the entire inside of the mask, including the tight folds and crevices were identified as probable worst-case locations where organic material would likely be present and could become entrapped. For this reason, residual bioburden measurements were sampled from the entire inside of the mask, including the tight folds and crevices.

For testing, a total of five Test BVM were purchased. Each mask was cut into two equal halves (Figure 1), thus producing two samples for analysis. The total inside surface area of each mask half was measured from a digital laser scan and measured 93.04 cm^2^. Each of the five decontamination protocols were repeated on two mask halves (n = 2).

### 2.2. Contaminants

A BVM is considered an oronasal mask typically covering a patient’s mouth and nose [28] and consequently, it may contact saliva, mucus, and microbial flora found in the upper respiratory tract. Many different bacteria can colonize the upper respiratory tract, and *Staphylococcus epidermidis* is among the most common to be found in the nasal and paranasal sinuses [29]. This gram-positive bacterial species was used in the current study to contaminate the Test BVM, as it is prevalent on human skin and most surfaces and forms a biofilm. This makes it a likely microorganism contaminating the BVM during use [30] and suitable for use in the current study.

Worst-case contamination conditions were achieved by fully submerging the Test BVM mask halves into a soil solution consisting of standard mucus test soil simulating mucus exposure from a cystic fibrosis patient [31] combined with *Staphylococcus epidermidis* ATCC 12228. A 2% transfer of *Staphylococcus epidermidis* ATCC 12228 stock culture to sterile Tryptic Soy Broth was prepared (1:49 dilution of culture to media) to obtain a 100 mL solution. The culture and media were then incubated overnight at 37°C.

The simulated mucus soil, termed Artificial Mucus Soil, was prepared according to an international standard for validation of cleaning methods for reusable medical devices [31]. The components of the Artificial Mucus Soil (mucin from pig mucosa, casein hydrolysate, sodium chloride, diethylene triaminepentaacetic acid, ASTM Water Type I, potassium chloride, salmon sperm DNA, freeze dried egg yolk emulsion, and phosphate buffered saline) were mixed on a stir plate at 20°C - 25°C to produce a uniform solution that provided protein, total organic carbon, nucleic acids, and carbohydrates as cleaning markers for the residual analyses. The overnight cultures were added to Artificial Mucus Soil at a concentration of 10% inoculum, which provided the addition of bacteria as a cleaning marker for the residual analysis. The Test BVM were fully immersed in the bacteria and Artificial Mucus Soil, allowed to incubate for 24 hours at 37°C, and set out to dry for 15 minutes before undergoing one of five reprocessing protocols.

### 2.3. Decontamination Protocols

Decontamination requires cleaning of the device to the point where visible bioburden is removed. According to FDA regulatory classifications, BVM are semi-critical reprocessed devices due to contact with mucous membranes (but not sterile tissue); therefore, the decontamination protocols for cleaning must remove visible bioburden and achieve high-level disinfection to eliminate microorganisms [1]. High level disinfection intends to kill vegetative bacteria, but does not eliminate all spores [32]. High-level disinfection requires a reduction of 6log_10_ in colony forming units (CFU) plus overkill as a measure of microorganisms in residual bioburden. A high log reduction value corresponds to an overall high bioburden removal as a result of cleaning and disinfection, which was the targeted goal for the experimental decontamination protocols in the current study.

**Figure 2.**
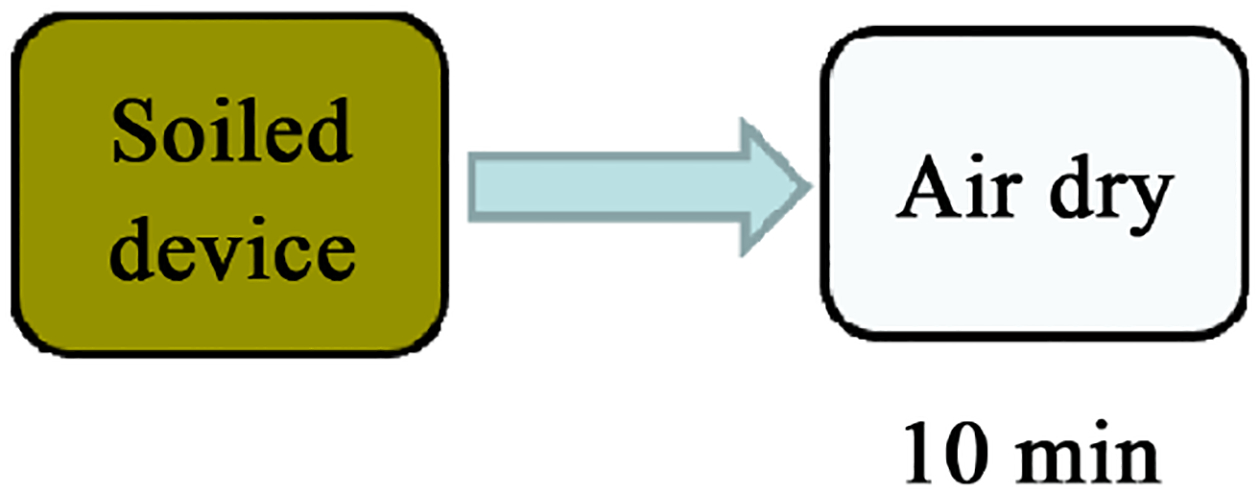
Negative control disinfection protocol.

**Figure 3.**
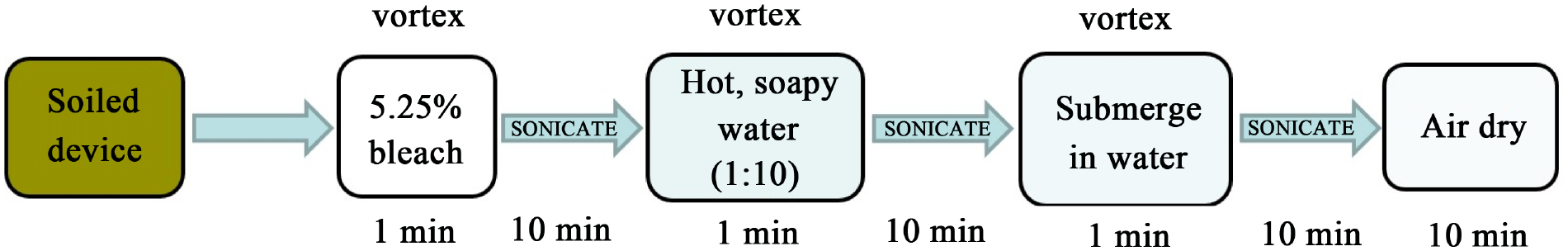
Positive control disinfection protocol.

Five decontamination protocols, including three experimental protocols and positive and negative controls, were applied to the Test BVM masks following contamination. The negative control was designed to yield a high bioburden and included masks that did not undergo any decontamination (Figure 2). The positive control was designed to eliminate all bioburden. The positive control consisted of submerging the BVM in full strength (5.25%) sodium hypochlorite solution (Clorox bleach, The Clorox Company, Oakland, CA), then hot (>40°C) water with non-enzymatic detergent (Versa-Clean™ Multi-Purpose Cleaner, Thermo Fisher Scientific, Waltham, MA), and lastly in filtered deionized water (ASTM type I) (Figure 3). For each protocol step, the mask was sealed in a container with the appropriate decontamination agents for that step and placed on a vortex mixer for 1 minute. Following this, the same mask and container were sonicated for 10 minutes before moving to the next decontamination agent.

**Figure 4.**
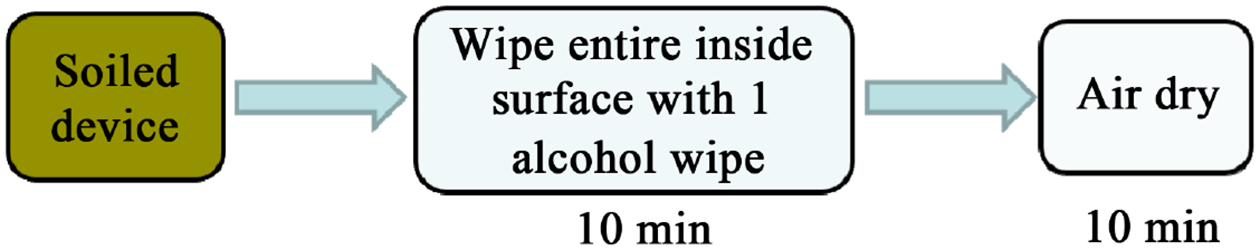
Alcohol wipe protocol.

**Figure 5.**
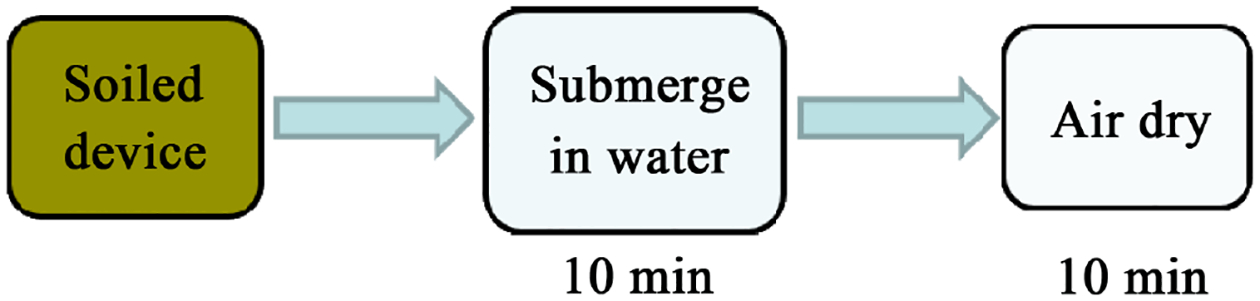
Water rinse protocol.

**Figure 6.**
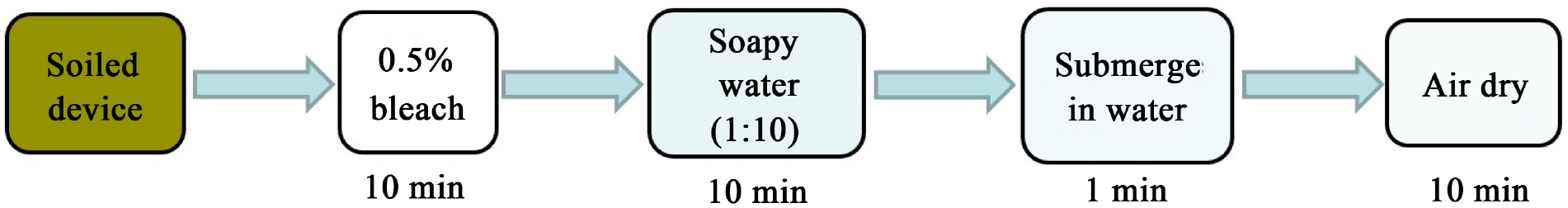
General disinfection protocol (soap and bleach). This soap and bleach protocol is currently in use at an urban Tanzanian hospital [10].

The three experimental decontamination protocols were chosen based on hospital reprocessing observations at three hospitals in Tanzania [10]. The Alcohol Wipe protocol involved wiping the entire inside of the mask with one 70% isopropyl alcohol wipe (Medium Sterile Alcohol Prep Pads (2.7 × 6.6 cm), Fisher HealthCare, China) (Figure 4). The Water Rinse protocol involved submerging the entire mask half in ASTM Type I water for 10 minutes (Figure 5). The Soap and Bleach protocol involved sequential 10-minute submersion of the mask half in a 0.5% sodium hypochlorite solution, a non-enzymatic detergent (1:10 dish soap to water), and ASTM Type I water (Figure 6). Following decontamination, each mask was air-dried for 10 minutes before being evaluated for residual bioburden.

### 2.4. Residual Bioburden Analysis

Three analytical methods were selected to evaluate the residual bioburden on the Test BVM following each decontamination protocol (Table 1). Sample collection for Metrics 1 and 3 involved swabbing the total inside surface area of each half BVM immediately after cleaning, except for the negative control cases that were swabbed 24 hours after contamination. Sample collection for Metric 2 involved swabbing approximately one-fourth of the inside surface area due to the manufacturer recommendation to use a small sampling area.

Metric 1 was a commercial method that provided a quick (~2 minutes), qualitative assessment of cleanliness by detecting the presence of residual carbohydrates, protein, and hemoglobin on sample swabs. Test strips provided by the manufacturer featured three colored pads that indicated the presence of physical bioburden in the swabbed region. According to the manufacturer, the detection limits for the test strips are ≥210 ug/ml for carbohydrate, ≥120 ug/ml for protein, and ≥0.25 ug/ml for hemoglobin. For this study, decontamination targets were met when both trials showed no indicator color change on any test strip pad, which was consistent with reduction of residual carbohydrates, protein, and hemoglobin below detection limits during decontamination.

Metric 2 was a commercial method that provided a quick (~10 seconds), quantitative assessment of cleanliness by detecting the presence of residual ATP on sample swabs. Reagent vials provided by the manufacturer emit bioluminescence, which correlates to certain ATP levels and was detectable as light emission when inserted into a handheld device also provided by the manufacturer. According to the manufacturer, the detection limit is 0.2 mg protein per swab, and a surface can be considered “clean” if the RLU (relative luminescence units) value displayed is less than 100. For this study, decontamination targets were met when both trials had RLU less than 100.

Metric 3 was a commonly utilized microbiological technique (standard plate count) that provided a quantitative assessment of disinfection by detecting the presence of residual bacteria on sample swabs, which present as CFUs on agar plates. Plate counts of CFUs were repeated in triplicate for swabs from each Test BVM half mask, averaged, and then divided by the plate dilution to obtain the concentration of bacteria. Overall log reduction of CFUs for a given decontamination protocol was calculated as the difference in bacterial concentration following the decontamination protocol (*C*_*P*_) relative to the negative control sample (*C*_*NC*_) (Equation (1.1)).

(1.1) $$\text{log reduction in bacteria}=\text{log}\left(C_{NC}\right)-\text{log}\left(C_{P}\right)$$

Sample collection involved swabbing the designated inside surface area of each half mask. Following instructions for use for Metric 1, swabs were placed into sterile test tubes with 10 ml of sterile Millipore (ASTM Type I) water and vortexed for 1 minute, followed by full immersion of the provided test strips into the solution and manual agitation for 10 seconds. The test strips were removed and held horizontally for 90 seconds prior to reading results. The test strips were compared to the color chart provided by the manufacturer, and the presence or absence of residue was recorded. Following instructions for use for Metric 2, swabs were placed in provided reagent vials and gently shaken for 3 seconds prior to reading results by inserting individual vials into the hand-held unit by the manufacturer and recording the displayed RLU value. Following standard microbiological methods for Metric 3, swabs were placed into sterile test tubes with 10 ml of sterile Millipore (ASTM Type I) water and vortexed for 1 minute. A ten-fold dilution series was prepared, plated onto agar (Tryptic Soy Agar, Remel, Lenexa, KS), and incubated for 24 hours at 37°C prior to reading results by manually counting CFUs for each plate.

## 3. RESULTS

### 3.1. Design Review and Impact on Decontamination

Careful review of the Test BVM identified several design features that were considered reprocessing challenges (Figure 1). The mask included contours on the outside surface, flaps of pliable materials, tight folds, and rounded cavities on the interior surface, all of which could become exposed to mucous and other biological contaminants from the patient while wearing the mask or during handling. The non-rebreathing valve had a complex geometry, with crevices and other small design features, dead-end chambers, and an in-line filter. If the valve remained assembled with the mask, the valve-mask interface would present an additional challenge in the form of a circumferential small crevice between the two parts. Upon disassembly from the mask, the tight fit of the modular connection may be difficult to reassemble and could be a site for potential failure after multiple reprocessing trials.

As anticipated from the design review, the BVM mask design features negatively impacted the performance of the decontamination protocols. Based on visual examination of the half masks, tight folds and rounded cavities on the interior surface of the mask retained more water and bioburden than other areas of the mask. This was most prevalent after the mask was removed from the soil solution and allowed to dry, when noted mask regions contained pools of the soil solution. Such areas were more difficult to reach during manual cleaning with the alcohol wipes compared to protocols based on mask submersion in cleaning solutions. This was reflected in the inconsistent results for the Alcohol Wipe protocol (Figure 4) using Metric 1 and the large inter-trial differences in the quantitative cleanliness values for Metric 2 and Metric 3 during the Alcohol Wipe protocol (Tables 2–4). For example, the Alcohol Wipe protocol reached 8log_10_ CFU reduction for trial 1, consistent with a high-level disinfection benchmark, but only reached 3log_10_ CFU reduction for trial 2. One possible reason for the inconsistency was inadequate wiping of the inner folds of the BVM mask during the second trial.

### 3.2. Residual Bioburden Analysis

All three methods for assessing residual bioburden required training of personnel based on instructions for use (Metrics 1 and Metrics 2) or standard microbiological techniques (Metric 3). Metric 1 required little training outside of the swabbing technique. However, Metric 1 was a qualitative assessment judged by the user performing the test and results were highly subjective due to differences in individual ability to identify a change in color. Metric 2 required ample training time for: 1) proper swabbing technique; 2) preparing the swab and reagent vial for insertion into the handheld device; and 3) operating the device. Metric 3 required time-intensive training for: 1) cell culturing techniques and use of lab equipment; 2) growing stock inoculum and culturing of bacteria; 3) proper inoculation of the BVM masks; and 4) growing bacterial samples on agar plates.

Based on these preliminary data, the Alcohol Wipe and Water Rinse protocols (Figure 4 and Figure 5, respectively) were ineffective or inconsistent at meeting decontamination targets. Overall, only the positive control protocol (Figure 3) met the decontamination targets for Metric 1 (Table 2) and only the Soap and Bleach (Figure 6) and positive control protocols met the decontamination targets for Metric 2 and Metric 3 (Table 3 and Table 4). The qualitative analysis used for Metric 1 had inter-trial variation, with indicators meeting cleanliness benchmarks (no color change) in trial 1 but not trial 2 for both the Alcohol Wipe and Soap and Bleach protocols. A color change was noticed for the hemoglobin indicator in the positive control protocol, but this was considered a false positive because of bleach present. The quantitative analysis used for Metric 2 and Metric 3 had large inter-trial differences for the Alcohol Wipe protocol, as mentioned above, but little inter-trial variations for the other protocols. For example, the RLU values for Metric 2 varied within the 0 – 100 RLU benchmark (e.g. 6 RLUs in Trial 2 for the Soap and Bleach protocol) but this is consistent with the sensitivity range of the system.

All three methods for assessing residual bioburden required some use of consumable materials and/or durable equipment. Metric 1 required use of consumable materials (swabs, test strips) to provide a qualitative assessment of residual bioburden (proteins, carbohydrates, hemoglobin) based on color change. Metric 2 required use of consumable materials (swabs, reagent vials) and durable equipment (refrigerator for reagent vials, hand-held device for measuring the RLU of emitted bioluminescence) to provide a quantitative assessment of residual ATP. Metric 3 required use of consumable materials (swabs, bacteria and growth media, agar plates, cell spreaders, pipette tips) and durable equipment (incubator, pipettes, biological hood) for measuring bacterial CFUs to provide a quantitative assessment of residual bacterial concentration.

## 4. DISCUSSION

This study completed a comprehensive design review of a disposable BVM to identify potential challenges to reprocessing and investigated three different methods for assessing residual bioburden on the BVM masks following decontamination. Overall, only the positive control protocol (Figure 3) met the decontamination targets for Metric 1 and only the Soap and Bleach (Figure 6) and positive control protocols met the decontamination targets for Metric 2 and Metric 3. The Alcohol Wipe and Water Rinse protocols (Figure 4 and Figure 5, respectively) were ineffective or inconsistent at meeting decontamination targets (Tables 2–4). These findings provide a first step toward development of device-specific protocols that define uniform reprocessing procedures and inspection criteria for hospitals choosing to reprocess BVM. Based on a small sample size, these preliminary results support the use of bleach-based decontamination protocols that submerge disposable BVMs into cleaning and disinfection solutions rather than wiping.

The BVM mask geometry negatively impacted the performance of the decontamination protocols. Flaps of pliable materials, tight folds and rounded cavities on the interior surface of the mask were challenging to clean and contributed to large variations in cleanliness for Metric 2 and Metric 3 in the Alcohol Wipe protocol. Although only the BVM mask was evaluated in the decontamination protocols, the design review identified additional challenges associated with the modular connection of the non-rebreathing valve, as well as the complex valve geometry having small crevices, dead-end chambers, and a filter.

All three methods required some level of training based on instructions for use (Metric 1 and Metric 2) or standard microbiological techniques (Metric 3). Metric 1 seemingly required the least training but generated the least consistent results. The time required for assessment after sample collection ranged from approximately 1 – 3 minutes for Metric 1 and Metric 2 and approximately 1 day for Metric 3.

Several drawbacks were noted for each of the residual bioburden assessment methods. Metric 1 required subjective interpretation of color change and was incompatible with protocols involving bleach, which induced a color change falsely indicating the presence of hemoglobin. This inaccuracy would be problematic if hemoglobin was a routine contaminant for a given SUD. Possibilities for overcoming color interpretation include use of a colorimeter to compare the test strip to the color standard or taking pictures of the test strips in a uniform, well-lit environment and completing hue analysis. Drawbacks for Method 2 and Method 3 center on the need for consumables and durable equipment and extended training time, as mentioned in the results.

Several study limitations are noted. These results may not be generalizable, as only one model of disposable BVM was evaluated in this study. This underscores the need for device-specific validation of reprocessing protocols, as design features vary between BVM and can impact the effectiveness of decontamination protocols. While the design review involved the entire Test BVM (full mask and non-rebreathing valve), the analytical methods for residual bioburden analysis (Metrics 1 – 3) were evaluated using masks halves (n = 2). This approach provided for efficient screening of the analytical methods, but it is recognized that additional evaluation of the full BVM (mask and valve) and statistical comparisons are needed for definitive conclusions related to the decontamination protocols. Finally, all analyses were performed by a team of five trained bioengineering graduate students with faculty supervision, which does not represent the personnel likely to conduct decontamination of disposable BVM in a healthcare setting.

## 5. CONCLUSION

This study describes an initial experimental approach for validating BVM decontamination protocols and generating data for objective assessment of reprocessing and reuse practices. The data support positive decontamination outcomes using the bleach-based in-hospital reprocessing protocol currently in use in some Tanzanian hospitals [10]. However, design features of the Test BVM mask presented clear challenges to cleaning and drying during the different decontamination protocols. Based on these preliminary results, continued assessment of the Soap and Bleach decontamination protocol (Figure 6) using complete disposable BVMs (full mask and the non-rebreathing valve connected to the mask) exposed to simulated use is warranted. Detailed inspection criteria, factors related to mask/valve assembly and disassembly, and the maximum number of intended reprocessing cycles, remain to be determined. Given proper consideration of training time and available resources, well-executed in-hospital reprocessing could support neonatal resuscitation strategies and other demands for manual resuscitation by helping to maintain adequate supplies of BVM.

## Figures and Tables

**Table 1.** Residual bioburden analyses.

Name	Method	Markers	Metric	Measure
Metric 1	^1^ChannelCheck™	carbohydrates, protein, hemoglobin	color change on test strip pads	qualitative
Metric 2	^2^ATP Complete®	adenosine triphosphate (ATP)	relative luminescence units (RLU)	quantitative
Metric 3	Standard Plate Count	bacterial growth	colony forming units (CFU)	quantitative

1 Healthmark Industries Company, Inc, Fraser, MI;

2 Ruhof Corporation, Mineola, NY.

**Table 2.** Metric 1 results.

Protocol	Metric 1						Target met?
	Protein Present?		Carbohydrates Present?		Hemoglobin Present?		
	Trial 1	Trial 2	Trial 1	Trial 2	Trial 1	Trial 2	
**Negative Control**	Yes	Yes	Yes	Yes	No	No	No
**Water Rinse**	No	Yes	Yes	Yes	No	No	No
**Alcohol Wipe**	No	No	No	Yes	No	No	No
**Soap and Bleach**	No	Yes	No	Yes	No	No	No
**Positive Control**	No	No	No	No	No	No	Yes

**Table 3.** Metric 2 results.

Protocol	Metric 2		Target met?
	ATP Value (RLU)		
	Trial 1	Trial 2	
**Negative Control**	9999	3967	No
**Water Rinse**	9999	7112	No
**Alcohol Wipe**	2343	8948	No
**Soap and Bleach**	0	6	Yes
**Positive Control**	0	0	Yes

**Table 4.** Metric 3 results.

Protocol	Metric 3		Target met?
	Log Reduction in Bacteria		
	Trial 1	Trial 2	
**Negative Control**	0	0	No
**Water Rinse**	3.7198	2.5179	No
**Alcohol Wipe**	8.2576	3.3010	No
**Soap and Bleach**	8.2576	7.1613	Yes
**Positive Control**	8.2576	7.1613	Yes

## ABBREVIATIONS

ASTM: American Society for Testing and Materials
SUD: Single-use devices
LMIC: Low- and middle-income countries
BVM: Bag-valve-mask(s)
DNA: Deoxyribonucleic acid
FDA: Food and Drug Administration
CFU: Colony-forming units
ATP: Adenosine triphosphate
RLU: Relative luminescence units
